# CRISPR-Cas and Its Wide-Ranging Applications: From Human Genome Editing to Environmental Implications, Technical Limitations, Hazards and Bioethical Issues

**DOI:** 10.3390/cells10050969

**Published:** 2021-04-21

**Authors:** Roberto Piergentili, Alessandro Del Rio, Fabrizio Signore, Federica Umani Ronchi, Enrico Marinelli, Simona Zaami

**Affiliations:** 1 Institute of Molecular Biology and Pathology, Italian National Research Council (CNR-IBPM), 00185 Rome, Italy; roberto.piergentili@cnr.it; 2 Department of Anatomical, Histological, Forensic, and Orthopedic Sciences, Sapienza University of Rome, 00161 Rome, Italy; federica.umanironchi@uniroma1.it (F.U.R.); enrico.marinelli@uniroma1.it (E.M.); simona.zaami@uniroma1.it (S.Z.); 3 Obstetrics and Gynecology Department, USL Roma2, Sant’Eugenio Hospital, 00144 Rome, Italy; fabrizio.signore@aslroma2.it

**Keywords:** CRISPR-Cas, germline genome editing, human embryo, bioethics, biosecurity

## Abstract

The CRISPR-Cas system is a powerful tool for in vivo editing the genome of most organisms, including man. During the years this technique has been applied in several fields, such as agriculture for crop upgrade and breeding including the creation of allergy-free foods, for eradicating pests, for the improvement of animal breeds, in the industry of bio-fuels and it can even be used as a basis for a cell-based recording apparatus. Possible applications in human health include the making of new medicines through the creation of genetically modified organisms, the treatment of viral infections, the control of pathogens, applications in clinical diagnostics and the cure of human genetic diseases, either caused by somatic (e.g., cancer) or inherited (mendelian disorders) mutations. One of the most divisive, possible uses of this system is the modification of human embryos, for the purpose of preventing or curing a human being before birth. However, the technology in this field is evolving faster than regulations and several concerns are raised by its enormous yet controversial potential. In this scenario, appropriate laws need to be issued and ethical guidelines must be developed, in order to properly assess advantages as well as risks of this approach. In this review, we summarize the potential of these genome editing techniques and their applications in human embryo treatment. We will analyze CRISPR-Cas limitations and the possible genome damage caused in the treated embryo. Finally, we will discuss how all this impacts the law, ethics and common sense.

## 1. Introduction

**Figure 1:**
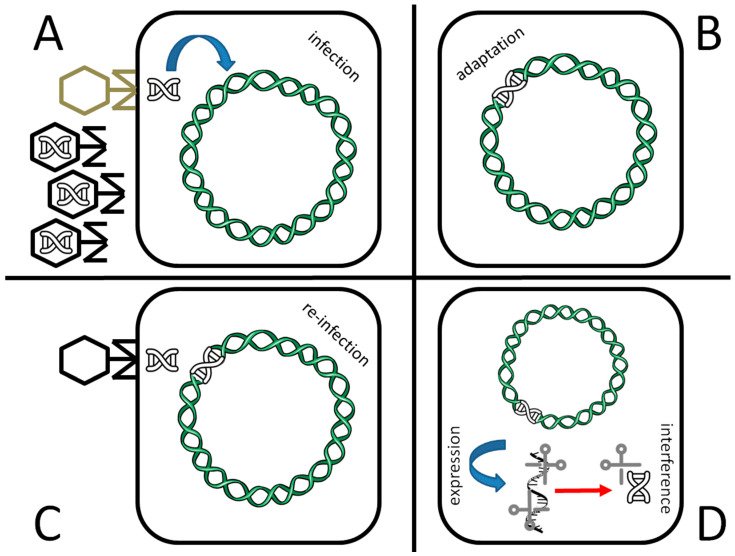
The CRISPR antiviral defense system of prokaryotes. A population of viruses may contain both wild type (black) and defective phages (gray; see text for further explanation). Upon infection of a defective bacteriophage as in the shown example (**A**), part of the viral genome is inserted inside one of the CRISPR loci of the bacterial genome (adaptation, **B**). In case of a second infection, even in case of a wild type phage (**C**) the CRISPR locus is transcribed (expression) and promotes viral genome degradation by site-specific, Cas-mediated cleavage (interference, **D**).

**Figure 2:**
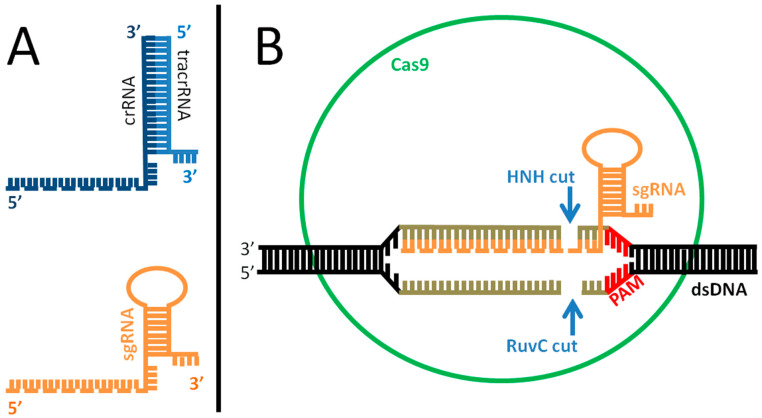
Mechanism of action of the Cas complex. (**A**): difference between natural (top) and engineered (bottom) guide RNA (gRNA). The natural system is composed of two parts, crRNA (dark blue) and tracrRNA (light blue), which are paired and drive Cas9 to target the invading viral DNA. The portion of the crRNA recognizing the target is indicated as a dotted line. In the engineered form, the synthetic, single guide RNA (sgRNA) is one molecule that mimics the shape of its natural counterpart, including the target recognition site (dotted line). (**B**): for genome editing, the sgRNA (orange) is incorporated inside the Cas9 protein (green) and recognizes the double-stranded target DNA (black-grey) promoting the pairing. In case of homology (pairing sequence length: 21–72 bp, indicated in gray) and in the presence of a PAM sequence (red) on the target DNA, the Cas protein cuts the DNA 3 bp upstream of PAM, causing a double strand break, thus inactivating target gene function. The repair of the target chromosome damage in eukaryotic cells usually employs the error prone NHEJ (non-homologous end joining) mechanism; however, in presence of an exogenous DNA template (injected with the Cas complex and sgRNA), the cell may fix the break through homologous-driven repair, thus introducing the sequence of interest inside the genome.

Although its first, serendipitous discovery dates back to 1987 [1], the potential of the CRISPR (Clustered Regularly Interspaced Short Palindromic Repeats) system for genome modification exploded less than 10 years ago [2], and earned Jennifer Doudna and Emmanuelle Charpentier the Nobel Prize in Chemistry in 2020 [3]. The CRISPR system is the way Bacteria and Archaea defend themselves from viral infections (Figure 1). It has been shown [4] that natural occurring, defective variants of bacteriophages that are still able to inject their genome inside the host but are unable to complete their life cycle, or are too slow in doing that, or even wild type phages that are in some way inactivated during their infection through other host defenses, are likely the principal route for bacteria to acquire virus resistance through the CRISPR system. In brief, if the host survives a first viral attack (Figure 1A), part of the phage genome may be incorporated inside a specific locus called CRISPR, composed of short DNA repeats (length: 23–55 base pairs, bp) [5], thus becoming a CRISPR DNA spacer (length: 21–72 bp) (Figure 1B). CRISPR loci are located at multiple sites inside the bacterial genome, (up to 23 in *Methanocaldococcus* sp.) and each locus may contain several spacers (up to ca. 600 spacers in *Haliangium ochraceum*) [6]. This part of the process is known as “adaptation”. If the bacterium is infected again by the same type of virus (Figure 1C), the CRISPR locus, which contains usually ca. 50 spacers per CRISPR array [5], is transcribed into a long RNA (CRISPR RNA, or crRNA) which is then cleaved into short interfering crRNA (Figure 1D) (process: “expression”). These pieces, together with the Cas protein and tracrRNA (see below), will bind to the newly injected viral genome, promoting its degradation through a nucleolytic cut (Figure 1D and Figure 2), a process known as “interference”. The protein(s) responsible for the pairing and cut is/are a member of the Cas (CRISPR-associated protein) family of proteins. They differ in their nucleic acid target (DNA or RNA; single or double stranded; linear or circular DNA; other structural features), the type of cut (blunt ends or overhangs) and the way of action; they are sorted in a total of two classes, and divided in six types and 33 subtypes [7]. The systems that use more than one Cas protein for DNA degradation belong to class I, while those belonging to class II use only one, larger Cas protein. Cas9 endonuclease, one of the most used for genome editing, belongs to class II. The Cas9 complex is formed by the Cas9 protein, which contains two magnesium-dependent endonuclease domains responsible for the DNA cutting (namely, HNH and RuvC) and two RNA, a crRNA and a trans-activating CRISPR RNA (tracrRNA), the latter being necessary for crRNA maturation and cleavage through the formation of a RNA duplex. Once processed by RNAse III, the complex crRNA/tracrRNA/Cas9 is “guided” to their target, and thus the name “guide RNA” (gRNA). A crucial role in the Cas9-mediated target degradation is played by the PAM (Protospacer Adjacent Motif) sequence. PAM is a short DNA string (usually 3–8 bp long, length and sequence depending on the bacterial species Cas9 comes from) adjacent to the cleavage site on the nontarget strand (Figure 2B). Usually, it is located 2–6 nucleotides at the 3′ end of the DNA sequence targeted by the guide RNA and the Cas nuclease cuts three nucleotides upstream of it. Notably, PAM is not present in the crRNA sequence (i.e., it is part of the viral, but not the bacterial, genome) and its absence is sufficient to impair the cleavage activity of the entire complex. This allows the complex to recognize self vs. non-self DNA, thus avoiding that it erroneously cuts bacterial DNA at the CRISPR locus. As such, the role of PAM is equally pivotal during genome engineering, for precise DNA targeting.

The work of Doudna and Charpentier was aimed at modifying this system to target specific genomic sequences in virtually any organisms and promote their cleavage; the modified system allows both gene knock out or knock in, depending on the repair mechanism involved. In particular, Doudna and Charpentier re-engineered Cas9 complex into a more controllable, two-component system by fusing the two RNA molecules into a synthetic, single guide RNA (sgRNA) that is sufficient to find and cut the target DNA of choice (Figure 2A, bottom), a solution explored and validated also by other groups in the same year [8]. In this way, it is possible to create in vitro a custom-made sgRNA that drives the Cas9 endonuclease to a specific target inside the genome. Once the sgRNA recognizes the homologue sequence, Cas9 cuts the DNA. This triggers the DNA repair machinery of the host cell, involving the error-prone NHEJ (non-homologous end joining) mechanism, which induces some errors in the joining ends, thus inactivating gene function (knock out). However, if a suitable exogenous template is provided (donor DNA), this can be used for a homology-driven DNA repair, thus substituting the original sequence with another one of choice, either (i) introducing a specific mutation in a wild type sequence (knock out), or (ii) restoring the wild type copy of a mutated gene (gene modification), or (iii) inserting an entire new gene or even multiple genes (knock in). Consequently, the donor DNA may potentially be of any size, ranging from a few base pairs targeting a gene point mutation, to larger elements containing one or more genes with specific promoters and additional regulatory elements. The relatively ease of use and the efficient and precise targeting of DNA sequences allowed to spread the use of this technique to modify genomic DNA in virtually all living organisms, including plants, animals and even humans [9].

### 1.1. Overview of the Possible Applications of CRISPR for the Improvement of the Human Quality of Life

The flexibility of the system opened up the possibility to modify host genome in very diverse ways, to achieve important results in both eukaryotic and prokaryotic organisms. Plant genomes can be easily modified with CRISPR [10]. In general, this technique allows to have crop improvement either for yield increase [11], better nutritional content [12,13], fruit ripening control [14,15], to create plants resistant to parasites, fungi and other pests [16,17,18,19] or resistant to environmental stresses [20,21]. In addition, also more “aesthetic” aims are pursued, such as more intense color, larger size or a more regular shape of fruits and vegetables, improved flowering of ornamental plants [22,23,24] or even the creation of vegetables with new flavors [25]. Some modifications also have a direct impact on human health; beyond the nutritional content mentioned above, there are groups working to lower the allergenic content of food, such as depleting allergens content of soybean [26] or to create low/free gluten wheat for coeliac people [27,28].

CRISPR had been used to manipulate animal genomes to create strains that are resistant to disease. This had been achieved for example for pigs resistant to Porcine Respiratory and Reproductive Syndrome virus (PRRSV) or the African swine fever or even to produce coronavirus resistant pigs, by manipulating the pig proteins the virus recognizes to invade cells. Pigs had been also manipulated to improve xenogeneic transplantation and tolerance [29,30]. Similar gene editing approaches had been used also for improving animal welfare after diffuse practices such as horn removal, male castration, or mulesing (reviewed in [31]). The poultry industry is deeply involved as well, for the creation of chicken and quail lines that are resistant to specific disease-causing microorganism such as avian influenza virus or avian leukosis virus; similarly, great efforts are in place for enhancing muscle growth, thus increasing the weight and quality of human food (reviewed in [32]). These animals are also used to create chickens that are efficient bioreactor systems for producing valuable proteins in poultry species (see [32] and references therein). Similarly to plants, also in animals CRISPR had been used for aesthetic purposes, such as the creation of ‘micropigs’ to be used as pets [33]; the same company also works on koi carps with custom size, color and patterns. Finally, also for animals, studies are running for allergen depletion in food; examples include goat milk [34] and chicken eggs [35].

As for direct research in human health, and beyond, its use in embryo manipulation that will be discussed in the next sections, we recall here briefly a few examples of CRISPR/Cas9 used as a tool to treat human diseases. This system is very useful to create disease models or to discover new etiological agents, allowing researchers to better understand their biology. Examples include cancer, neurological diseases, cardiovascular diseases, immunodeficiency, infectious diseases, sickle cell disease, hemophilia, metabolic diseases, cystic fibrosis, retinitis pigmentosa, and several others (reviewed in [36,37,38,39,40,41,42]). In particular, the treatment and characterization of cancer is very promising, and hundreds of publications are available on this topic (for recent reviews, see for example [43,44,45,46,47,48,49,50,51]). Pavani and collaborators showed its use in the treatment of beta-thalassemia [52] and there are ongoing projects for the treatment of AIDS [53]. Several works exist describing a possible CRISPR-based approach in the treatment of liver diseases such as viral hepatitis, hepatocellular carcinoma and hereditary tyrosinemia type I (reviewed in [54]). This system has also been used to specifically remove entire chromosomes from the genome. Zuo and collaborators [55] demonstrated that it is possible to specifically remove sex chromosomes from mouse cultured cells, embryos, and tissues in vivo, and also eliminate from mouse cells human chromosomes that are otherwise stably transmitted through generations, such as chromosomes 14 and 21; this is also possible in human cultured cells, such as aneuploid cancer cells or cells from Down’s syndrome patients. This work [55] suggests the potential use of CRISPR/Cas9 as a therapeutic strategy for human aneuploidy diseases caused by supernumerary chromosomes.

We conclude this section by citing also work aimed at eradicating human, pest-derived diseases such as malaria, by trying to control their vectors, i.e., mosquitoes [56]. This is a typical case of artificial ‘gene drive’, i.e., the possibility to spread a particular gene (or a set of genes) in a population by altering its probability of transmission through the generations. The potential of this technique has been greatly enhanced by the use of CRISPR, thanks to the possibility, in heterozygotes, to target (cut) the wild type gene and then repair it using the mutated copy as a template; as a consequence, the probability to transmit the mutated gene to the offspring is nearly 100%, instead of 50% as expected according to Mendel’s laws. This approach overcomes the problems of inserting a particular mutation inside a genome and waiting for it to naturally expand (if natural selection does not swipe it away), and allows to genetically modify specific populations or even entire species. However, this raises a plethora of ethical issues about the possibility to permanently modify the genome of the target species worldwide, influence its ecology and that of the related species, the impossibility to foresee all possible side effects of the manipulation of the targeted gene, and possibly causing species extinction [57,58].

### 1.2. Technical Risks of Human Embryo Modifications

First of all, the fundamental difference needs to be stressed between modifying a human embryo, for research purposes, and then discarding it after data collection, and allowing its implantation. Both are highly controversial topics, but there is a general consensus in opposition to the latter possibility. Laws currently in force, although still insufficiently implemented, ban this practice in most countries.

The first report of CRISPR-Cas driven editing of human embryos is only five years old [59], indicating that this approach for gene therapy is just in its infancy, as someone already noted [60,61]. Despite this, CRISPR potential and ease of use suggest that human gene editing could become a possible way to treat—at least some of the—human genetic diseases, either inherited (Mendelian diseases) or caused by newly appeared mutations (as in idiopathic cancer) at the individual level, towards the implementation of precision medicine [62]. This can be achieved in two ways: by deleting a wild type (wt), target gene that predisposes to a disease or infection, or by substituting a mutated gene with its wt counterpart. The efficiency of both approaches heavily depend on the number of somatic cells that can be edited, thus the idea of manipulating the zygote or the embryo at a very early stage, being much lower the number of cells to be targeted, or to act on gametes to significantly reduce mosaicism [63]. An example of the first approach, used in embryos, is the very debated experiment by the Chinese biophysicist He Jiankui and his staff, aimed at preventing HIV infections by inducing a random deletion in an otherwise wt protein coding gene (namely, CCR5)—we already described how this story ended [64]; moreover, the several theoretical, technical and methodological pitfalls of those experiments had been extensively analyzed in the past [65]. Obviously, most efforts are based on the second method, i.e., restoring the wt sequence. The ethical and legal issues of using these technologies in human embryo modifications will be extensively discussed in the following sections and the possibility to modify the method to avoid this change to pass on the next generation (“one-generation germline therapy”) has been discussed as well [66]. In particular, the one-generation germline therapy is promising because it would allow to treat an inherited condition in the somatic line leaving the germ line almost untouched. In brief, it consists of introducing not just one gene template, but a more complex transgene cassette hosting the following modules: (i) the gene of interest under the control of a selected promoter (for example, the wild type copy of a mutated gene under its physiological promoter); (ii) a DNA recombinase (such as Cre) under the control of a germ line specific promoter; (iii) specific flanking sequences, recognized by the recombinase; (iv) an insulator (a cis-acting DNA sequence) that avoids the unwanted activation of the recombinase promoter in the soma, under the influence of the upstream promoter. In the soma, the gene of interest is activated under the stimuli specific for the promoter of choice, while the recombinase gene remains silent and the cassette is stably inserted in the genome and passed through cell divisions. Instead, in the germ line, the promoter of the recombinase is activated, and the protein promotes the cleavage of the entire cassette at the flanking sequences (recognition sites), stimulating the DNA repair machinery that would restore chromosome integrity but without the cassette and with only a small DNA footprint, i.e., a few additional DNA nucleotides at the site of repaired cleavage. The possibility to insert the cassette in a specific location where the footprint would (likely) cause no health problem is, obviously, critical.

Beyond possible, subtle mutations that can be a byproduct of off-target Cas9 action, and the possibility to induce chromosome alterations in model embryo systems [67,68] and in human somatic cells [69,70], potential risks for whole genome integrity had been highlighted in at least three reports published in 2020, indicating that large and unexpected karyotype modifications can occur, causing potentially severe health problems, that might arise during CRISPR use in human embryo treatment. The first report we discuss is the one by Alanis-Lobato and collaborators [71]. On the basis of previous work, suggesting that gene editing is occurring through interhomologue homologous recombination (IH-HR) driven by the maternal allele [63], the authors tried to replicate the experiments using as a target the pluripotency factor OCT4, encoded by the *POU5F1* gene on the p-arm of chromosome 6. Despite the overall good results (i.e., restoration of the wt allele), the authors did not obtain the PCR amplification of the supposedly edited gene. An in-depth analysis revealed that almost one third of the targeted samples (8/25) showed abnormalities on chromosome 6. In particular, the authors were able to identify diverse rearrangements, including segmental loss or gain of chromosome fragments next to the *POU5F1* locus (p-arm), whole gain of chromosome 6, and segmental gain on the q-arm, totaling approximately 16% of samples and spanning 4 kb to at least 20 kb in length. In addition, also the search for loss of heterozygosity (LOH) was positive. In the second paper, Zuccaro and collaborators [72] reported the results of substituting the *EYS* locus (mapping at 6q12, associated with retinitis pigmentosa and causing blindness, [73]) in embryos. The starting point was the use of spermatozoa from a man homozygous for an intragenic deletion causing the formation of a premature stop codon at exon 34 of the gene, exploiting the fact that DSB (double strand break) mediated recombination between homologous chromosomes in eggs is apparently quite efficient and preferentially uses the maternal allele as a template [63]. Indeed, Zuccaro and collaborators found that 17 out of 20 analyzed samples had the restoration of the wt genotype, suggesting either cell type differences in DSB repair and/or cell survival after a chromosome break. To better understand what happened, the authors performed several additional analyses, finding that in many cases the wt homozygous genotype for *EYS* was achieved with a contemporary LOH of the surrounding regions of chromosome 6. This LOH was caused by different gross chromosome 6 segmental rearrangements, including (i) distal 6q arm loss; (ii) 6q arm gain (with the consequent increase of paternal genes copy number) and a contemporary movement of the *EYS* gene far from flanking sequences or with breakpoints inside the EYS locus, resulting in the impossibility to amplify and detect it in the samples; (iii) monosomy of the maternal chromosome 6, i.e., complete loss of the paternal homologue; (iv) gain of one or more paternal chromosome 6. This occurred in embryos either injected with Cas9 RNP at fertilization or at the 2-cell stage, in 19 out of 20 samples screened. In conclusion, the authors wrote that in their system—i.e., very early stage of development—the loss of paternal alleles is a ‘common outcome’ and that this occurs through aneuploidy, not efficient interhomolog repair. Interestingly, additional chromosome aberrations and aneuploidies involving chromosome 16 were scored, mostly in the form of mosaic; at least some of them are due to off-target CRISPR-mediated cleavage. All together, these results suggest that in human preimplantation embryos DNA repair pathways might be—or behave—different(ly) from other cell types (in [72] there is evidence of the involvement of the MMEJ—microhomology-mediated end joining—pathway [74]), which could explain the high incidence of endogenous genome instability. The third paper we discuss is by Liang and collaborators [75] in which no karyotype alteration is presented, yet extensive gene conversion is reported as a consequence of CRISPR-mediated DNA damage. Additionally, in this case the authors performed experiments on preimplantation heterozygous embryos and using a heterozygous mutation in exon 22 of the *MYH7* gene located on chromosome 14, implicated in familial hypertrophic cardiomyopathy (HCM). The authors noted that DNA damage in this system is mainly repaired through gene conversion, a form of HDR (homology directed repair) that uses an exogenous homologous sequence as a template for repairing. Interestingly, HDR is usually far less used in cells than NHEJ, also because it can occur only after DNA replication while the other may act throughout the cell cycle. The main difference between classical HDR and gene conversion is that, after strand annealing, the repair machinery may extend the copy of the template beyond the microhomology region used for the first strand invasion, thus making the template and the invaded strand identical (noncrossover recombination), and consequently causing a LOH that might extend for several kilobases and, in some instances, entire chromosome arms. In addition, the search for chromosome deletions in those cells was negative, confirming the gene conversion mechanism involved in this experiment and indicating that ‘a large percentage of DSBs (41.7%, 50/120), are resolved by gene conversion’ [75]. Similar results were obtained targeting the *MYBPC3* and *LDLRAP1* loci [75].

Despite the different results obtained by Liang and collaborators [75] compared to the previous two [69,70], i.e., LOH as main output but no chromosome deletion vs. karyotype alterations including deletions, duplications and chromosome loss, all three works point to the same conclusions: (i) embryos respond differently to DNA damage when compared to somatic cells upon CRISPR-Cas9 manipulation, and the reasons of this difference are still far from clarified; (ii) the output of this manipulation is still largely unpredictable and amply variable, including several different types of genomic damage; (iii) in all cases, the percentage of damaged cells is extremely high—around half of them show gross alterations. For all such reasons, we believe that using in embryos the same protocols used for somatic cell editing is presently inadvisable. Taken together, these results are against the use of CRISPR-Cas9 for manipulating embryo genomes because of the formation of DSBs that might either remain unrepaired or being erroneously repaired, through mechanisms that are still poorly understood, and the extensive LOH that might reveal additional recessive mutations inside the egg genome. It would be advisable to opt for alternative gene editing methods that do not cause DNA damage [76]; nonetheless, even that approach needs further testing and verification.

## 2. Beyond Therapeutic Safety and Efficacy, Genome Editing Entails Polarizing Ethical and Legal Quandaries

Ethical concerns about CRISPR-based genome engineering techniques arise from various lines of reasoning. Firstly, the scope and limitations of CRISPR technology, including the risks stemming from limited on-target editing efficiency, mosaicism [77], and inaccurate on- or off-target editing, are still largely unknown [78]. Such flaws have been documented in CRISPR experiments with animals as well as human cell lines. Still, as the technology is gradually honed and perfected, such concerns may no longer be warranted over time. Nonetheless, ethically tenable decision making in biomedicine needs to be informed on an empirical basis, by means of a thorough appraisal of risk–benefit ratios. To that end, ethical decisions have to be grounded in a thorough analysis of possible outcomes, the likelihood of each manifesting itself, and the purposes and possible justifications that determine the end results. When it comes to CRISPR genome engineering technology, however, assessing potential risks and benefits with an acceptable degree of accuracy may be extremely hard, given the difficulties of making reliable predictions about the future of a genetically edited organism. It is unclear whether modified organisms will be affected indefinitely and whether and to what extent the edited genes will be transferred to future generations, potentially affecting them in unexpected ways. An accurate risk–benefit analysis is therefore significantly complicated, not only by technical limitations, but also on account of the complexities inherent to biological systems.

### 2.1. Current Lack of Understanding Stands in the Way of thorough Risk–Benefit Analysis

Critics have in fact pointed out that even if genome-editing procedures are successfully carried out, and the expected functional effect is achieved in a timely fashion, genetic information and biological phenotypes are related in ways that are not yet fully understood. Genetic pleiotropic effects in fact constitute a primary source of phenotypic variability in humans. It is therefore still undetermined in what way CRISPR-Cas systems could bring about pleiotropic effects, even though we were able to successfully establish that genes act as prominent causal factors in disease development. Some disease phenotypes, it is worth stressing, can potentially be altered or even obliterated by pleiotropic effects [79]. Hence, editing a gene in germline and/or somatic cells may bring about unpredictable biological consequences. It is in fact the complex regulatory actions of numerous genes which determine a wide array of biological traits. That makes it extremely difficult to “engineer” a biological phenotype at the level of a whole organism [80]. In other words, a single gene is very unlikely to be the only factor that molds and develops a complex biological trait. The emergence of a biological phenotype is in fact determined by environmental and epigenetic factors and several other genetic regulatory factors, e.g., additional genes or distal regulatory elements (such as enhancer or repressor elements). By virtue of that, a thorough understanding of other independent variables contributing to the phenotype’s instantiation is a key element, in addition to genetic modification. Such an understanding is nevertheless still far from being complete enough in many normal and disease processes. Since uncertainty still lingers as to how gene expression and modification affect and drive complex biological outcomes, a thorough risk–benefit analysis is difficult to produce [81]. In addition, as previously noted, a seemingly unsolvable ethical and legal debate has been ignited by the potential application of CRISPR technology on human embryos [82]. Such a controversy is further complicated by the lack of consensus among ethicists and legal scholars as to the status of the human embryo itself [83,84]. Even though many in the scientific community contend that experimentation on human embryos after 14 days is ethically intolerable, it is all but impossible to find common ground for determining the status of a human embryo and when it acquires “personhood” [85,86]. That is the fundamental reason why many nations regulate medically assisted procreation via in vitro fertilization with varying degrees of restrictions [87].

### 2.2. The Unsolved Quandary of Embryonic Status

If embryos are to be ascribed personhood status, then they are entitled to have their inalienable human rights upheld. If, on the other hand, they are deemed as something in between, i.e., less than human beings but more than mere pools of cells, what moral rights should they have acknowledged, if any? Certainly, some point out that the first experiments using CRISPR to edit human embryos occurred in 2015, and since then, only few teams around the world have focused on the process and its potential [85], but recent studies have highlighted an underappreciated risk of CRISPR–Cas9 editing: if embryos are deemed to have the right to at least some degree of legal safeguards, such safety concerns are likely to significantly inform the ongoing debate on the matter. In light of such major unsolved controversies, some have called for an international moratorium on all embryo editing [88,89], and some countries, including Canada, already have policies that ban human-embryo gene editing, irrespective of whether or not the edited embryo would be meant for implantation [90]. In the United States and Britain, on the other hand, an intermediate regulatory approach has been chosen. The US Food and Drug Administration views any use of CRISPR/Cas9 gene editing in humans as gene therapy, regulated by the FDA’s Center for Biologics Evaluation and Research (CBER). Clinical studies of gene therapy in human beings therefore require the submission of an investigational new drug application (IND) before they can be legally initiated in the United States. In addition, marketing gene therapy products calls for the submission and approval of a biologics license application (BLA). As a result of such requirements and restrictions, operating a private lab, with private funds, and conducting nonclinical, human gene therapy research is not illegal. Nonetheless, marketing such therapeutic options in the US would require FDA approval in terms of clinical studies and marketing. As far as it could be determined, no instances exist of a germline gene therapy product in the US; only somatic cell gene therapy products have been granted approval; currently, federal law prevents the FDA from reviewing or approving any application involving manipulated human embryos [91]. Again, it is necessary to draw a clear distinction between embryo editing for research purposes and the implantation of such edited embryos, which is ethically far more contentious. In the United Kingdom, for instance, the use of genome editing in embryos for the purpose of implantation is banned, albeit gene editing on discarded IVF embryos is lawful, provided that such embryos are destroyed immediately afterwards. In vitro culture of human embryos beyond 14 days after onset of embryo creation, i.e., after the appearance of the primitive streak, is prohibited: such a ban is enshrined in the Human Fertilisation and Embryology (HFE) Acts of 1990 and 2008 [92]. In Italy, specific provisions in law 40/2004 recognize the embryo as having rights from the moment of fertilization [93]. The law prohibits the use of embryos for any research unless it is specifically aimed towards improving the therapeutic and medical condition of the embryo itself [94,95]. Critics have pointed out the apparent paradox behind such a restriction, considering that in vivo embryos can be terminated up to 24 weeks through voluntary termination of pregnancy [96]. Some may in fact find it confusing that embryo research is required to stop so much earlier, particularly in light of the fact that it is arguably more ethically sustainable to use abandoned supernumerary embryos for research purposes that could benefit humanity, than to just dispose of them. While abortion ethics is beyond the scope of this review, it is worth pointing out that in these two scenarios, different fundamental goals are in play: legal termination of pregnancy stems from the need to uphold the right of women to have a choice and be in control of their body, whereas in vitro embryo research does not entail that issue. As for embryo experimentation, such intermediate regulatory approaches bear witness to the current uncertainty as to how strictly such techniques ought to be regulated, for the purpose of striking a balance between upholding bioethics precepts and fostering scientific progress for the common good. Nonetheless, nations with more lax, ambiguous or nonspecific regulatory frameworks governing new biomedical technologies may result in a worrisome “maverick” scientific environment in which untested techniques are made available. That has been found to be the case with mitochondrial replacement therapy (MRT), a form of nuclear transfer used as a germline therapy and believed to prevent the transmission of mitochondrial diseases and increase the likelihood of success in pregnancies [97]. Although MRT is banned in many countries due to its still dubious safety, clinics in Spain, Albania, Russia, Ukraine, and Israel have been found to offer the procedure [98]. That said, and irrespective of how individual countries decide to govern such techniques, the issue of whether scientists should seek to edit human embryos to prevent genetic diseases is controversial in itself, because the genomic change which it creates is permanent and may be passed down for generations. Even if embryo experimentation should be deemed justified, by virtue of its potential benefit to the embryo itself and others, embryos obviously cannot grant informed consent, but are still liable to experience life-altering consequences which can extend throughout their lifetimes and affect future generations as well. Besides, as mentioned earlier on, the enforcement and practice of both ethical precepts and legal provisions are inextricably linked to a set of notions that are hardly carved in stone and universally acknowledged. Hence, the time at which a human life (whether embryo or fetus) is deemed a fully-fledged human being has far-reaching ramifications that encompass the crucial realms of health care, law- and policy making and the inalienable right of individual autonomy of all humans [99]. There are no easy answers in our ever more culturally and ethically diverse societies: one-size-fits-all approaches seem doomed to fail, yet finding common ground is vital. If human embryos are to be deemed human beings with full personhood status, major implications ensue. That is the perspective espoused by Catholic doctrine, best exemplified by the late Pope John Paul II, who in 1995 famously stated that “the mere probability that a human person is involved would suffice to justify an absolutely clear prohibition of any intervention aimed at killing a human embryo” [100]. That approach does not differentiate between embryos edited for research purposes and edited embryos to be implanted. Conversely, prominent philosophers such as Kant, Locke and Fletcher have laid out criteria for identifying personhood closely tied to self-awareness, the capability to relate to others, self-control, rationality, and the use of memory, among others [101,102,103,104]. On the other hand, all such complexities and apparently irreconcilable views notwithstanding, there is no denying that banning or constraining research on human embryos could put a damper on scientific progress and stymie the development of therapies that could defeat currently untreatable diseases. Would that not be a moral and ethical imperative outweighing previously reported concerns? Again, no easy answers.

### 2.3. Broadening Fields of Application

Early research seeking treatment for neurodegenerative diseases has shown that CRISPR can even be used in combination with other elements, e.g., nonpathogenic viruses, to help improve target specificity on any genomic sequences [105,106]. CRISPR-Cas9 may also be pooled with multiple guide RNAs, allowing the editing of multiple genes in one step. This pool of guide RNAs allows the Cas enzyme, which cuts the DNA, to be guided to many different parts of the genome. It could prove valuable to target multiple genes at once through pooled guide RNAs in order to shed light on systemic effects, e.g., response to therapy or how metabolism is affected [107,108]. Another ethically sensitive research field that holds great promise involves the investigation and development of the therapeutic potentials of stem cells and the biology of totipotent cells, which can divide indefinitely and give rise to any of the 220 cell types found in an embryo as well as extra-embryonic cells (i.e., the placenta) and pluripotent cells (which can give rise to all cell types of the body except for the placenta) [109,110]. Totipotent and pluripotent cells are in fact not found in any viable human tissue sources other than embryos. In fact, embryonic cells within the first couple of cell divisions after fertilization are the only cells that are totipotent, derived from the early cells of a fertilized egg, while pluripotent cells are found in the inner cell mass of blastocysts [111,112]. In the current pandemic scenario, studies have found that CRISPR/Cas9 has potential applications to human-induced pluripotent stem cells (hiPSCs), ranging from gene therapy to the induction of the immunological response to specific virus infection, such as HIV and SARS-Cov-2 itself [113]. The potential applications of CRISPR/Cas9 and hiPSCs in antiviral response, including SARS-Cov-2 research, are centered around a testing platform meant to replicate the human lung, differentiating wild type (WT)-hiPSCs into pneumocytes type II [114], and treating them with pseudoviruses capable of replicating SARS-Cov-2 infection [115]. Although future prospects for therapeutic applications are still far from conclusive, especially as far as mutation-prone viruses such as SARS-Cov-2 are concerned, that may certainly be one way in which gene editing could be harnessed for the repression or the upregulation of genes that play a role in viral activity, in addition to the introduction of polymorphisms that could protect against or predispose to the viral infection [116]. Moreover, cells that have undergone editing can be used to test the capacity of a number of compounds to fight the infections. A rather versatile and apparently effective approach has recently been devised for the purpose of targeting viral RNA through CRISPR/Cas9; researchers are looking into the possibility of specifically using it on SARS-CoV-2 RNA genome, in order to constrain its ability to reproduce. That prospect could pave the way for a great opportunity to effectively deal with fast-evolving viruses that have the capability to develop resistance rapidly and give rise to tragic consequences such as overwhelmed health care systems and ethical quandaries in the delivery of care [117,118]. In light of the above instances of therapeutic applications, it is not hard to figure out why it is so essential to strike a balance between bioethics precepts and the needs and fast-developing dynamics of scientific research, for the sake of public health. On the other hand, several roadblocks still lie on the path of techniques such as the Prophylactic Antiviral CRISPR in huMAN cells (PAC-MAN). Research has shown that such methods could inhibit RNA viruses in human cells through CRISPR-Cas13d for the purpose of viral gene expression inhibition through targeted RNA degradation. Difficulties in that approach include the lack of a reliably effective delivery mechanism, since the component parts of CRISPR are just too large to enter the target cells. Progress is in fact being made in terms of developing lipitoids, i.e., synthetic molecules to be used as CRISPR delivery system [119]. Still, clinically viable CRISPR-based potential therapeutic options such as PAC-MAN [120] may still be far from mainstream therapeutic use, in terms of accuracy, applicability and costs, hardly feasible solutions for the current scenario.

## 3. When the Line between “Therapy” and “Enhancement” Is Blurred

Provided that genome editing techniques undoubtedly have therapeutic value and hold great promise in terms of providing new forms of treatment for incurable diseases, prominent bioethicists have advocated for it by pointing out that they may even be ethically desirable [121,122]. In fact, gene-editing technologies could even result in fewer embryos being destroyed in assisted reproduction procedures. At the time being, if a carrier of a genetic disease seeks to have a child who will not have the same condition, the carrier often resorts to IVF and preimplantation genetic diagnosis (PGD) for the purpose of selecting an embryo unaffected by that condition. Still, it behooves us to bear in mind that IVF often entails producing a considerable number of supernumerary unwanted embryos, which will eventually be destroyed even if viable. This ethically objectionable practice would be obsolete if safe and effective gene-editing technologies were available to all; carriers of genetic diseases in fact would no longer have to produce large numbers of surplus embryos in order to make it possible to have children not affected by the genetic condition of which their parents are carriers, e.g., autosomal recessive mutations [123], responsible for autosomal recessive monogenic diseases such as cystic fibrosis and sickle cells anemia [124,125]. The feasibility of such prospective applications has, however, been called into question, given the potential for harm, off-target effects or mis-edits that could make any such theoretical advantages ultimately unattainable [126]. Plus, it is unlikely for gene editing to replace PGD, since embryo testing will always be necessary, as will embryo selection. Testing will in fact still have to be performed following the editing intervention, to minimize the risks arising from the adverse effects of germline genome editing. Hence, as long as editing techniques are still quite far from 100% accurate, embryos might again be discarded even after the intervention [127]. As the United States National Academy of Science (NAS) stated in a report issued in 2020, in fact, neither the currently available editing technologies, nor sequencing embryonic DNA, aimed at keeping in check possible off-target effects, are as yet reliable enough, hence strict criteria ought to be met for couples to avail themselves of such techniques in order to have biological children that will not inherit the mutation. Key factors include the nature or severity of the disease, awareness of correlations between genotype–phenotype, and the availability of already mentioned alternative options, such as prenatal genetic testing (PGT) and noninvasive genetic diagnosis (NIPT) [128]. Not all possible applications of heritable human germline editing can rely on responsible translation pathways, hence a thorough risk–benefit analysis rests on multiple complex variables from which an assessment has to be worked out: disease severity, the genetic situation of the couple, the mode of inheritance of the disease, the nature of the proposed sequence change, and the availability of alternatives [129]. Outside of its therapeutic potential, however, an exhaustive discussion as to the ethics of gene editing cannot leave out the all too relevant aspect of possible applications aimed at biomedical enhancement. Currently, the primary means for the enhancement of cognitive functions and/or physical performances is pharmacological in nature. Pharmacological cognitive enhancement refers to the off-label use of drugs or supplements by healthy individuals in order to augment performance. Such drugs are referred to as nootropics or “smart drugs”. Nootropic use is universally and increasingly popular, both by professionals and students, in addition to recreational users [130,131,132]. Among the most widespread substances are methylphenidate [133], fluoxetine, and sildenafil [134,135]. Pharmacological enhancers already pose a major and immediate challenge for regulators and policy makers, which would only be compounded if the use of genome editing techniques were used to enhance the capabilities of human beings yet to be born. Hence, through such interventions, individual traits and characteristics could be selected during its gestation according to the wishes and decisions of third parties. That would amount to a sort of “germline genetic enhancement”, i.e., overcoming the limitations of the human body and mind, going well beyond the therapeutic use meant to restore or sustain health [136]. Even though the dream (or according to opponents, the nightmare) of being in full control of human biology might still be far off, gene editing research has already made giant strides [137,138]. Critics have expressed concerns that this approach would generate the need for a reflection on the ethical, social and legal implications of these techniques and their implementation in society. Fears that scientists may one day start “playing God” are not new after all: decades ago, in vitro fertilization (IVF) has been harshly criticized by many as “unnatural” or taking on prerogatives that do not belong to men [139,140,141]. IVF techniques are still morally and ethically controversial and often restricted, as are fertility preservation procedures capable of prolonging the time frame in which parenthood is achievable [142,143,144,145].

### 3.1. Is Enhancement Headed down a Dangerous Path?

Despite the potentially enormous benefits such techniques may yield, detractors have gone so far as to liken gene editing-based enhancement to eugenics: enhancement is after all an intervention aimed at improving capabilities, functioning or appearance that are already within the normal range. Eugenics was steeped in utilitarian philosophy precepts based on Darwinian natural selection. By virtue of that, eugenicists encouraged those deemed “fit”, typically belonging to middle and upper classes to have large families, whereas the destitutes, deemed “unfit”, were to breed less. Over the 20th century, it became clear that adjudging moral worth on the basis of mental or physical fitness would lead to atrocities of horrific magnitude, such as forced sterilization, euthanasia and genocide [146]. Although from the late 19th century eugenics was advocated for in Western countries, from the United States (suffice it to say that the Rockefeller, Carnegie, and Ford Foundations actively funded eugenics research) to the United Kingdom, Germany and Sweden, such ethically and scientifically indefensible thinking was ultimately exposed as folly, and rightly rejected. Nowadays, however, prominent supporters of human enhancement include academics and philosophers who argue that the aspiration to augment human capabilities, even at the genetic level, is grounded on solid science, individual consent and determination to improve oneself, thus devoid of any element of coercion [147], and that even a moral obligation exists to produce the best possible children [148]. Those are the underpinnings of the so-called Principle of Procreative Beneficence (PPB), a philosophically complex and contentious framework [149], which should in our view be rejected, since it would entail ascribing degrees of human value on the basis of capabilities or “desirable” attributes and quite possibly, placing lower moral value on the disabled or on those less endowed [150,151,152]. The alleged “obligation” to pursue such objectives is an integral part of the transhumanist doctrine, whose followers have espoused nontherapeutic gene editing and other new and emerging technologies for enhancement purposes [153]. In fact, transhumanists have embraced the desirability and inevitability of germline and enhancing gene therapies, while also calling for public financing of research and a regulatory process to ensure their safety [154]. Aside from extremes such as the notion of a “morally obligatory” enhancement of those yet to be born, three aspects should in our view be defined and specified when weighing the ethical feasibility of genome editing applied to reproductive technologies: the already mentioned moral status of embryos that should be enhanced, the legal status of the individual poised to be enhanced, and the responsibility of the agents carrying out the enhancement interventions. It is in fact undeniable that genetic enhancement can impact inalienable human rights such as identity, dignity, and good lifetime of individuals particularly vulnerable and without autonomy, such as embryos or a newborn [155]. Ultimately, as remarked in the Second International Summit on Human Genome Editing [156], the risks and benefits of germline genome editing are not fully understood and clarified yet, at least not enough to allow germline genome editing to proceed. That being said, expanding scientific understanding and recent research seem to point out that it may be time to start outlining what a well-balanced pathway for future clinical use should entail [157]. Mapping out the way forward would call for greater awareness, further discussion and consensus building on multiple highly complex issues; yet, those issues need to be thoroughly dealt with, since over time, the definition of a tenable and consistent clinical pathway for germline genome editing could become both morally essential and necessary to prevent irresponsible practices that would violate both clinical ethics and the core values on which our civilization is built.

### 3.2. Gene Drives: A Potential Threat to the Environment and Ecosystems?

As previously mentioned, in addition to its human therapeutic applications, CRISPR has already been used to modify animals [158], insects [159], plants [160], and microorganisms [161]. Successful trials [162] have also involved bacterial pathogens, such as the rather recent characterization of a new type 2 CRISPR system from *Francisella novicida* (FnCas9), an unofficial fourth subspecies of the *Francisella tularensis*, the causative agent of tularemia. Specifically, FnCas9 has been found to direct homology-directed repair (HDR) as well as nonhomologous end-joining–mediated DNA repair, while giving rise to a higher rate of HDR and extremely low off-targeting. A substantially high degree of specificity has been shown in terms of binding to its intended targets, which demonstrates that the horizon of genome editing technologies is poised to broaden considerably further [163]. Although such uses may not appear to create new ethical problems in such contexts, a real risk does exist that CRISPR could run counter to regulations governing the creation and release of genetically modified organisms (GMOs). Generating modified organisms could be valuable in terms of possibly eradicating infectious diseases through the elimination of their vectors and invasive species [164]. In 2014, Esvelt and coworkers suggested that CRISPR/Cas9 could be harnessed to build endonuclease gene drives [165], and studies documenting the successful engineering of CRISPR-based gene drives in Saccharomyces [166], Drosophila [167], and mosquitoes [168] were published soon after. Inheritance distortion has been found by all four studies, efficient over future generations. More recent experiments demonstrate that a CRISPR/Cas9-based gene drive can spread a targeted gene throughout nearly all of laboratory populations of yeast, fruit flies, or mosquitoes [169]. Among the noteworthy examples of such research have involved the *Aedes aegypti* mosquito, which spreads dengue fever, and even some *Anopheles* subspecies [170,171], which carry the *Plasmodium* parasite [172,173]. Biotech researchers have been resorting to “gene drives”, aimed at stemming disease transmission through the editing of mosquitos for the ultimate purpose of making them incapable of carrying the disease, inducing sterility in male mosquitos, or shortening the lifespan of their offspring [174]. Although such methods could prove extremely valuable in eradicating deadly diseases (malaria has struck 228 million people in 2018, causing upwards of 400,000 deaths [175]), environmental concerns have been expressed that they could cause the extinction of entire species, with harmful environmental consequences. Gene drive is a powerful tool that can make the edited trait heritable. Hence, genetically modified organisms which are released into the environment find wild-type mates, and their offspring is estimated to have a 50% chance of inheriting the modified genes [176]. Nonetheless, such fears (and hopes) appear to be overblown, being largely based on anecdotal, speculative theories, rather than solid empirical research and analysis. The progress in such techniques notwithstanding, there is still no proving how dangerous or promising they may turn out. While the introduction of small numbers of edited mosquitos or other pests is unlikely to cause major effects, gene drive is capable of copying mutations made on one chromosome by CRISPR to its partner chromosome, thus passing the edited genome on to future generations [177]. Although those dynamics could be instrumental in greatly limiting transmission rates of various infectious diseases, gene drives do entail considerable risks to the environment and ecosystems: they have in fact the potential to decimate entire species [178,179], disrupting food chains, or lead to the uncontrolled proliferation and spread of invasive species, without sufficient containment mechanisms [180,181,182]. It is therefore of utmost importance to identify the knowledge gaps that could thwart or complicate attempts to control CRISPR-modified species, as there may be unintended effects such as altering gene flow within a population [183]. Even though attempts have been made to model gene drives within populations [184], it is quite hard for such assessments to encompass ecosystems rife with complexities and extremely dynamic [185,186]. In order to face such risks and potential threats to ecosystems and biodiversity, research has been looking into ways to make gene drives reversible, through self-exhausting forms of CRISPR-based gene drive that have been dubbed “daisy-chain drive” [187,188], in order to achieve reversible alterations, meant to be limited in space and scope [189].

### 3.3. Should CRISPR Be Viewed as a Potential Biosecurity Hazard?

In 2017, the US Defense Advanced Research Projects Agency (DARPA)’s Safe Genes programme announced the allocation of USD 65 million to fund research into how to control, counter and reverse gene drives [190,191]. The program has been devised to pursue three fundamental technical goals: developing technologies for spatial, temporal, and reversible control of genome editors applied to living organisms; molecular countermeasures aimed at creating viable prophylactic and treatment solutions to stave off or constrain genome editing in organisms and preserve genomic backgrounds in populations; the capability to root out undesired edited genes from ecosystems, thus restoring them to their genetic baseline state. It is stressed that the development of these powerful techniques has to unfold and progress within a strict ethical framework which prioritizes safety and progress achieved in a responsible fashion [192]. DARPA program manager for the Safe Genes program has likened the measures and technologies to control, inhibit, or reverse genome editing to “brakes in cars”, which are not meant to make people drive slowly, but rather to enable them to drive fast and stop when they need to. The manager went on to remark that “the steep drop in the costs of genomic sequencing and gene editing toolkits, along with the increasing accessibility of this technology”, has engendered even greater opportunity to experiment with genetic modifications. In addition, this convergence of low cost and high availability means that applications for gene editing, both positive and negative, could arise from individuals or state entities that operate outside the scope and supervision of the traditional scientific community [193]. The ethical implications of such remarks are quite startling, in light of the potential such techniques indisputably possess. In fact, CRISPR can be accurately viewed as a dual-use technology: although it has a broad range of potential benefits to medicine, science, and public health, it can be used with malicious intent. As pointed out in a 2018 report [194] by the United States National Academies of Sciences, Engineering, and Medicine, requested by the U.S. Department of Defense, it is critical that biotechnology continue to be researched and developed, but at the same time and just as importantly, its innovative applications in the scientific and public realms ought to be thoroughly evaluated via a specifically targeted assessment framework. Possible risks were also identified in the report such as the re-creation of known pathogens (e.g., smallpox) as one of the gravest concerns, whereas the creation of a new pathogen was deemed a lower risk. As a matter of fact, CRISPR could make it possible to edit a given pathogen so that it will acquire the degree of virulence of another pathogen, or it might enable rogue scientists to replicate a known pathogen whose genome has been published. In light of those areas of concern, the misuse of CRISPR deserves to be acknowledged as having the potential to pose a real threat to biosecurity. How effective such oversight efforts at the national level will prove remains to be seen. Certainly, there are no silver bullet solutions that can be put in place in order to lower the risks of misuse to near zero [195]. Awareness of that fact makes it even clearer just how urgent the need is for targeted, globally shared regulations meant to define standards for the testing and environmental release of GMOs. Such innovative applications are in fact inadequately governed by current national and international regulations, particularly in terms of guidance and oversight. Those shortcomings are likely to induce public mistrust in the safety and viability of gene editing and GMOs, as well as in the regulatory bodies and agencies charged with overseeing such techniques. One of the main concerns in that respect is that public misunderstanding and mistrust of GMOs will hinder scientific progress and the highly beneficial applications of CRISPR. Thoroughly weighing, planning—and above all, getting right—the regulations and research ethics for these applications of CRISPR could go a long way towards creating an ethical framework for human germ line editing that can be shared at least by nations with common core values.

### 3.4. Towards a Shared Regulatory Framework?

A common set of standards to be applied to gene editing is therefore more essential than ever and needs to be based on the awareness that research ethics needs to be based on a painstaking process of gaining informed consent from those who will be individually impacted by a given procedure. Still, that plain and indisputable standard is not easily applied to gene editing and gene drive research, which are designed to modify entities yet to be born or even environments and ecosystems. The cornerstone of ethics-based research and innovation in those realms must therefore be based on fundamental values applicable to categories of research in the broad sense, in keeping with the fundamental precepts enshrined in human rights treaties and conventions. The very essence of human rights undoubtedly comprises both the duty of nations to uphold the people’s rights, in terms of physical well-being and autonomy, and to enable them to rely on a wholesome living environment. It is after all the very notion of human dignity on which the United Nations Universal Declaration of Human Rights is based [196]. Guidelines and recommendations by national and international scientific institutions must be drawn upon as valuable frame of reference, for the purpose of updating the moral and ethical compass through which genome editing is regulated. To that end, the already mentioned recent analysis by the NAS has outlined valuable guidance for the gradual and supervised development of the most controversial form of gene editing: heritable human genome editing [129]. Specifically, dramatic and highly consequential breakthroughs need to be regulated by prioritizing safety. That of course calls for an extremely cautious selection of relatively few initial cases, based on a thoroughly implemented risk–benefit analysis, and initial outcomes must be reviewed and evaluated before further procedures are given the nod. Moreover, when a high degree of uncertainty exists as to the procedure’s efficacy, interventions need to be focused on patients suffering from clinical conditions with high mortality and/or morbidity and for which no alternatives are available. Abiding by such standards would be in keeping with the most accurate assessment of potential harms versus benefits, and could ensure that human applications of such techniques are offered after an ethically tenable informed consent process. The NAS also calls attention to two ethical issues that further buttress those three standards: as mentioned earlier, heritable human genome editing affects the future existence of individuals who would be created as a result of the technology, but obviously cannot provide consent, which can be granted by their parents only. Secondly, germline editing gives rise to genetic alterations which can be inherited by future generations. Those points make the need for shared solutions and criteria all the more urgent, however hard it may be to achieve it. That perspective had already been highlighted by three reports issued by the World Health Organization Expert Advisory Committee on Developing Global Standards for Governance and Oversight of Human Genome Editing, a global multidisciplinary expert panel that set out to delve into the scientific, ethical, social and legal challenges linked to human genome editing, whether somatic or germ cell [197]. The WHO Committee, which held its first meeting from 18–19 March 2019, pointed out that relatively few countries have as yet outlined and codified an appropriate translational pathway for somatic human genome editing interventions, relying on well-balanced and enforceable regulation and oversight mechanisms aimed at patient safety and public confidence. Hence, looking forward, the priority set by the WHO’s expert working group was to gain an understanding and awareness of how to best foster transparency and reliable practices, and to make sure that any decision and authorization will be based on appropriate risk/benefit assessments, in apparent agreement with the NAS considerations.

## 4. Conclusions: When Technology Outpaces Regulatory Governance, Harmonization May Be Key

Like all potentially revolutionary technologies that seem poised to call into question core values that are deeply held in our societies, genome editing is bound to entail daunting quandaries. Ethical complexities and apparently irreconcilable elements need to be dealt with through balanced legal and regulatory approaches aimed at upholding the rights of all parties involved and no less importantly, fostering public health in the face of extraordinarily challenging circumstances. That prospect has to rely on a broad-ranging risk–benefit analysis. This is currently difficult to achieve, considering that several international laws severely restrict or ban such research or prevent it from being adequately funded. Reliable data concerning benefits and risks are largely unavailable. It is of utmost importance that governments reconsider their reasoning for putting in place such restrictions, so as to ensure that they are really warranted and not merely rooted in fear. At the same time, CRISPR applications to non-human organisms cannot be overlooked in their potential to pose biothreats. The extraordinary significance of germline alterations for individuals and societies has not yet been publicly debated. Yet, a multidisciplinary discourse could reliably and efficiently enable policymakers, funders, research institutions, and users to draw distinctions between appropriate applications of a technology from those and those that are inappropriate, intolerable, or even dangerous. Going forward, many support establishing an organization that will decide how best to address the aforementioned ethical complexities. Initiatives such as the International Summit on Human Genome Editing are a step toward the ultimate goal of finding middle ground and shared solutions at least among nations founded on a common framework of core values.

## Data Availability

The data presented in this study are available on request from the corresponding author.
